# Targeting MCL-1/BCL-X_L_ Forestalls the Acquisition of Resistance to ABT-199 in Acute Myeloid Leukemia

**DOI:** 10.1038/srep27696

**Published:** 2016-06-10

**Authors:** Kevin H. Lin, Peter S. Winter, Abigail Xie, Cullen Roth, Colin A. Martz, Elizabeth M. Stein, Gray R. Anderson, Jennifer P. Tingley, Kris C. Wood

**Affiliations:** 1 Department of Pharmacology and Cancer Biology, Duke University, Durham, NC 27710, USA; 2 Program in Genetics and Genomics, Duke University, Durham, NC 27710, USA

## Abstract

ABT-199, a potent and selective small-molecule antagonist of BCL-2, is being clinically vetted as pharmacotherapy for the treatment of acute myeloid leukemia (AML). However, given that prolonged monotherapy tends to beget resistance, we sought to investigate the means by which resistance to ABT-199 might arise in AML and the extent to which those mechanisms might be preempted. Here we used a pathway-activating genetic screen to nominate MCL-1 and BCL-X_L_ as potential nodes of resistance. We then characterized a panel of ABT-199-resistant myeloid leukemia cell lines derived through chronic exposure to ABT-199 and found that acquired drug resistance is indeed driven by the upregulation of MCL-1 and BCL-X_L_. By targeting MCL-1 and BCL-X_L_, resistant AML cell lines could be resensitized to ABT-199. Further, preemptively targeting MCL-1 and/or BCL-X_L_ alongside administration of ABT-199 was capable of delaying or forestalling the acquisition of drug resistance. Collectively, these data suggest that in AML, (1) the selection of initial therapy dynamically templates the landscape of acquired resistance via modulation of MCL-1/BCL-X_L_ and (2) appropriate selection of initial therapy may delay or altogether forestall the acquisition of resistance to ABT-199.

Acute myeloid leukemia (AML) is a hematopoietic malignancy defined by clonal expansion of myeloid precursors. Among the molecular characteristics that typify this cancer, several studies have highlighted the dependence of AML cells on the anti-apoptotic protein BCL-2 and subsequently established how that specific dependency can be exploited for therapeutic effect using BH3 mimetics^1^,^2^, a class of compounds that affords direct inhibition of anti-apoptotic BCL-2 family members^3^. Of these agents, ABT-737, a BH3 mimetic that antagonizes BCL-2, BCL-X_L_, and BCL-w, demonstrated remarkable single-agent efficacy against AML in preclinical studies^2^. However, the clinical translatability of its orally available counterpart, ABT-263, has been limited due to dose-dependent thrombocytopenia secondary to BCL-X_L_ antagonism^4^. A second agent, ABT-199, sidesteps this limitation through specific inhibition of BCL-2^1^,^5^; it recently completed phase II clinical trials for the treatment of relapsed/refractory AML with promising results^6^. Given their clinical potential, many groups have reported mechanisms of resistance to ABT-737 and ABT-199 in myeloid and lymphoid malignancies^2^,^7^,^8^,^9^. Nevertheless, it remains unknown how focused antagonism of BCL-2 by ABT-199 will shape the landscape of acquired resistance in AML. In this study, we characterize ABT-199-resistant cell lines generated through chronic drug exposure to implicate BCL-X_L_ and MCL-1 as the main mediators of resistance to ABT-199 in AML and demonstrate that combinatorial inhibition of BCL-2/BCL-X_L_/MCL-1 can be used to delay or altogether forestall the acquisition of cell-autonomous drug resistance.

## Results

### Pathway-Activating Screen Nominates MCL-1 and BCL-X_L_ as Mediators of Resistance to ABT-199

**Figure 1:**
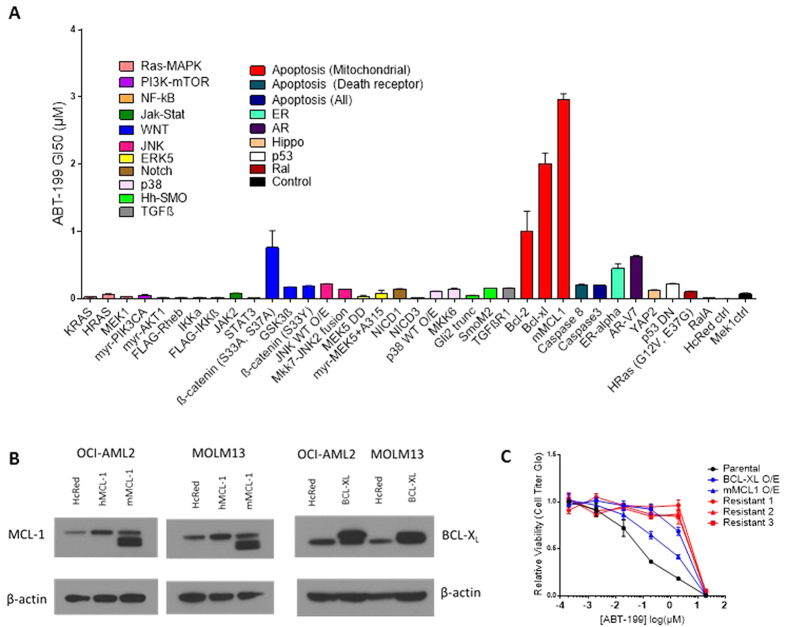
Pathway-Activating Screen Nominates MCL-1 and BCL-X_L_ as Mediators of Resistance to ABT-199. (**A**) Discrete populations of MOLM-13 cells were individually transduced with 37 pathway-activating cDNA constructs (Table S1). Drug sensitivity of each population was evaluated with a GI_50_ assay; data shown are mean GI_50_ (μM) ± SEM. Screen data from OCI-AML2 cells can be found in supplemental data (Fig. S1). (**B**) Western blot analysis of OCI-AML2 and MOLM13 lines overexpressing BCL-X_L_ and mMCL-1. hMCL-1 refers to overexpression of human MCL-1 (40 kDa) while mMCL-1 denotes overexpression of a murine MCL-1 (35 kDa) construct with mutated ubiquitination sites to enable overexpression. mMCL-1 was used in the screen due to concerns about rapid hMCL-1 degradation. HcRed is a negative control construct. Immunoblots shown are representative of three independent experiments. (**C**) ABT-199 dose-response curves for parental OCI-AML2 cells, derivatives resulting from overexpression (O/E) of BCL-X_L_ or MCL-1, or derivatives resulting from selection in the presence of chronic drug exposure (Resistant 1–3). Viability data is expressed as a percentage of DMSO-treated cells. SEM is of three independent experiments and indicated by error bars.

In order to identify signaling pathways whose activation is sufficient to impart resistance to ABT-199, we infected discrete populations of OCI-AML2 and MOLM-13 cells with constructs from a published lentiviral cDNA library encoding constitutive activators of 17 major oncogenic growth and survival pathways (Supplementary Table S1)^10^. These two cell lines were chosen for their sensitivity to ABT-199, with IC_50_ values previously reported to be below 10 nM^1^. The relative sensitivity of each of these isogenic cell line derivatives to ABT-199 was successively evaluated using an eight-point GI_50_ assay (Figs 1A and S1). Remarkably, activating the vast majority of the surveyed pathways conferred little to no resistance to ABT-199 or produced differing results across the two cell lines screened. However, stable overexpression of MCL-1 and BCL-X_L_ (Fig. 1B) yielded GI_50_ values greater than 10- and 20- fold higher than control, respectively, in both OCI-AML2 and MOLM-13 cell lines.

### Acute Myeloid Leukemia Cell Lines Acquire Resistance to ABT-199 Following Chronic Exposure

To determine whether AML cells would naturally acquire resistance to ABT-199 through modulation of MCL-1 and BCL-X_L_, we established a panel of resistant cell lines by exposing six AML cell lines to increasing doses of ABT-199 over several months. The following AML cell lines were selected and represent a range of baseline sensitivities to ABT-199, in order of decreasing sensitivity: HL-60, MOLM-13, OCI-AML2, THP-1, NOMO-1, and OCI-AML3; OCI-AML3 was known to be intrinsically resistant to ABT-199, with a baseline GI_50_ above 1 μM. Drug doses were initiated at each cell line’s GI_50_ to ABT-199 and increased upon stabilization of cell viability, up to a final dose of at least 2.50 μM ABT-199. In side-by-side comparisons of dose response curves to ABT-199, resistant derivatives were shown to have GI50 values up to 100-fold higher than matched parental lines (Fig. 1C). Complementary measurements of drug-induced apoptosis with flow cytometry using FITC-conjugated Annexin V in parental and resistant OCI-AML2 and THP-1 cells indicated significantly higher induction of apoptosis in parental cell lines in response to 48-hour incubation with ABT-199 (Fig. S2).

### BH3 Profiling Reveals an Acquired Dependence on MCL-1 and BCL-X_L_ in ABT-199-Resistant Cells

**Figure 2:**
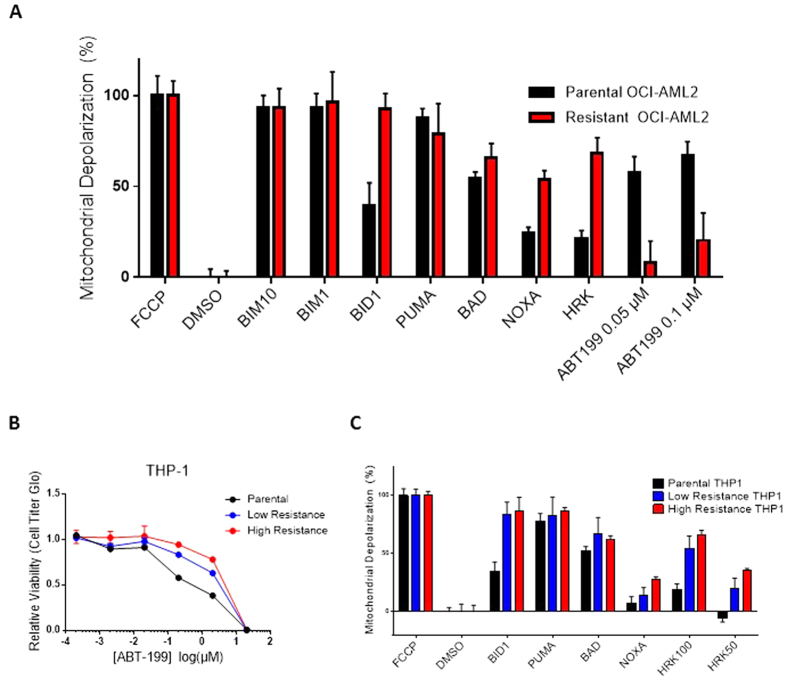
BH3 Profiling Reveals an Acquired Dependence on MCL-1 and BCL-X_L_ in ABT-199-Resistant Cells. (**A**) BH3 profiling of parental and evolved-resistant OCI-AML2 cells reveals increased mitochondrial depolarization in resistant OCI-AML2 lines in response to NOXA and HRK peptides (indicated in red). Percent depolarization shown here is calculated as the area under the curve normalized to carbonyl cyanide p-trifluoromethoxyphenylhydrazone (FCCP), the positive depolarization control. Dimethyl sulfoxide (DMSO) is a negative control. Unless otherwise indicated, the peptide concentration used was 100 μM. ABT-199 was applied as a molecular probe at the concentrations indicated. Data shown represent the mean ± SD of three independent experiments per peptide. (**B**) ABT-199 dose-response curves for parental and differentially resistant THP-1 cell lines. Viability data is expressed as a percentage of DMSO-treated cells. SEM is of three independent experiments and indicated by error bars. (**C**) BH3 profiling of parental and differentially resistant THP-1 cell lines, performed as described above.

We credentialed each of the parental and resistant cell lines using BH3 profiling. Briefly, this assay involves permeabilization of the cell membrane followed by staining with a mitochondrial potential-sensitive dye, and acquaintance of exposed mitochondria with peptides representing the functional BH3 domains of BH3-only proteins^11^,^12^. Known binding affinities between pro- and anti- apoptotic BCL-2 family members are used to infer the relative dependencies of parental and resistant cell lines on different anti-apoptotic proteins. Across all cell lines tested, mitochondria from parental lines and their resistant derivatives were found to be equivalently primed for apoptosis, as evidenced by comparable depolarization induced by the PUMA peptide (Figs 2A and S3). However, in each parental-resistant pair we observed substantially more mitochondrial depolarization in resistant lines upon exposure to peptides from HRK (which preferentially binds BCL-X_L_) and/or NOXA (which preferentially binds MCL-1), indicating a corresponding shift in dependency (Figs 2A and S3). Similar shifts were also observed in OCI-AML2 cells overexpressing BCL-X_L_ or MCL-1 (Fig. S4). While no proapoptotic activator singly binds BCL-2, we can approximate the contribution of BCL-2 by subtracting the HRK signal from the BAD signal. Using this approximation, we observed, in the resistant cell lines, a departure from BCL-2 dependence concomitant to their newly formed dependence on BCL-X_L_/MCL-1. This idea is further evidenced by decreased depolarization induced by direct application of ABT-199 in resistant versus parental cells (Fig. 2A). Note that the concentrations of ABT-199 used in this assay are substantially lower than their relevant concentrations in culture; this is because the profiling assay involves membrane permeabilization and therefore permits direct interaction between drug and mitochondria.

While evolving THP-1 cells to resistance, we intentionally preserved a “low resistance” line by maintaining a population at a sub-maximal dose (1.5 μM ABT-199) while scaling up the dose (to 2.5 μM ABT-199) of a separate population to produce a “high resistance” line. Characterization of the parental and differentially resistant THP-1 lines suggests that (1) the degree of drug resistance acquired is proportional to the background dose of drug (Fig. 2B) and that (2) the relative antiapoptotic dependence, as measured by BH3 profiling, of resistant lines shifts away from BCL-2 and toward BCL-X_L_/MCL-1 as they become more resistant (Fig. 2C).

### Upregulation of MCL-1/BCL-X_L_ Accompanies Acquired Resistance to ABT-199

**Figure 3:**
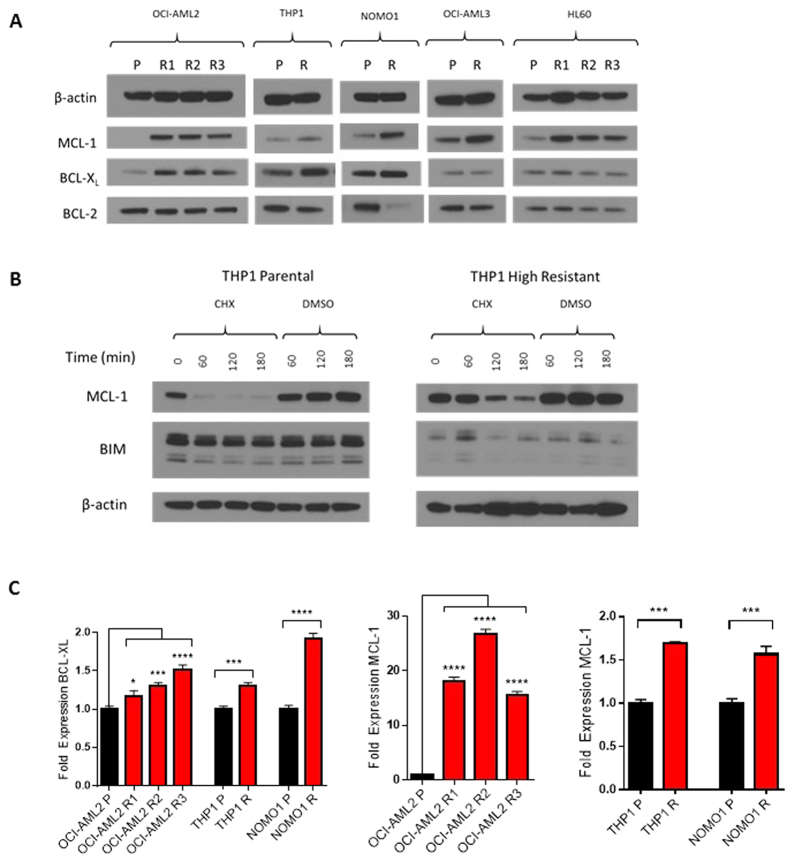
Upregulation of MCL-1/BCL-X_L_ Accompanies Acquired Resistance to ABT-199. (**A**) Western blot analysis of paired parental (P) and evolved resistant (R) cell lines immunoblotted as shown. R1, R2, and R3 refer to discrete populations of OCI-AML2 cells separately evolved to resistance. Blots are representative of three replicate experiments. (**B**) Parental and “high resistance” THP-1 lines were treated with 20 μg/mL cycloheximide (CHX) or DMSO for specified time intervals. After treatment, whole-cell lysates were made and analyzed by immunoblot. Blots shown are representative of three independent experiments. (**C**) qRT-PCR analysis of MCL-1 and BCL-X_L_ in parental (black bars) versus resistant derivatives (red bars) of OCI-AML2, THP-1, and NOMO-1 cell lines. Data are means ± SD from three experiments. *p < 0.05, ***p < 0.001, ****p < 0.0001 by Student’s t-test for THP-1 and NOMO-1, by Dunnett’s multiple comparison test for OCI-AML2 derivatives relative to parental line.

Subsequently, we examined whether the shift in anti-apoptotic dependencies suggested by BH3 profiling could be substantiated by changes in BCL-2 family protein expression. Western blot analysis revealed increases in MCL-1 and/or BCL-X_L_ in resistant versus parental lines (Fig. 3A). In some lines, this shift was accompanied by downregulation of BCL-2. These findings comport with our functional BH3 profiling data and indicate a collective shift in the cellular anti-apoptotic balance away from BCL-2 and toward MCL-1/BCL-X_L_.

Next, we investigated protein stability and transcriptional upregulation as potential causes of increased MCL-1 and BCL-X_L_. We treated sensitive and resistant THP-1 cells with cycloheximide, harvested treated cells at 1 hour time points, and analyzed for MCL-1 quantity by western blot. Under cycloheximide treatment, we observed substantial maintenance of MCL-1 through three hours in the resistant line while the MCL-1 signal was lost by one hour in the parental line (Fig. 3B). Parallel investigations regarding BCL-X_L_ were unsuccessful due to a half-life greater than 24 s. Quantitative real-time polymerase chain reaction (qRT-PCR) revealed a modest 1.4–1.6 fold increase in MCL-1 transcript levels in resistant THP-1 and NOMO-1 lines relative to parental (Fig. 3C). We observed greater-than 15-fold increases in MCL-1 transcript abundance in each of three resistant OCI-AML2 lines, likely inflated by low parental MCL-1 levels (Fig. 3A). In each of THP-1, NOMO-1, and OCI-AML2, we observed 1.2–1.6 fold increases in BCL-X_L_ transcript levels relative to parental.

We then sought to determine whether the increased dependence on BCL-X_L_/MCL-1 is induced acutely upon exposure to ABT-199 or gradually selected for as resistance is acquired. We exposed parental OCI-AML2 and THP-1 cells to a fixed concentration of 1 μM ABT-199 and collected samples at interval time points through 48 hours. Western blot analysis shows an acute and sustained increase of MCL-1 within two hours of drug treatment, while protein levels of the other BH3 family members remain unchanged through 48 hours (Fig. S5). We observed no acute changes in MCL-1 transcript levels (data not shown), implying that the acute increase in MCL-1 by 2 hours may be due to increased protein stabilization.

### Targeting BCL-X_L_/MCL-1 Resensitizes ABT-199-Resistant AML Cells and Delays Onset of Acquired Resistance to ABT-199

**Figure 4:**
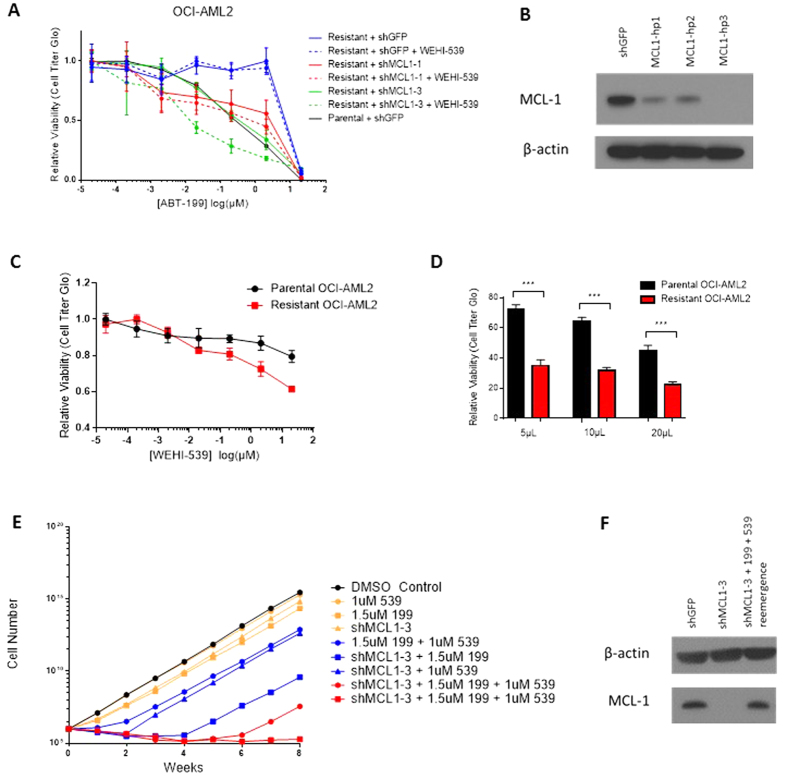
Targeting BCL-X_L_/MCL-1 Resensitizes ABT-199-Resistant AML Cells or Delays Onset of Acquired Resistance to ABT-199. (**A**) ABT-199 dose-response curves for an evolved ABT-199-resistant OCI-AML2 line and subsequent resensitization of that line using the BCL-X_L_ inhibitor WEHI-539 and/or either of two independent hairpins targeting MCL-1 (Table 1). Viability data is expressed as a percentage of DMSO-treated cells. SEM is of three independent experiments and indicated by error bars. (**B**) Immunoblot demonstrating hairpin knockdown of MCL-1 in evolved ABT-199-resistant OCI-AML2. Blots are representative of three replicate experiments. (**C**) WEHI-539 dose-response curves for parental and ABT-199-resistant OCI-AML2 cell lines. The resistant OCI-AML2 derivative used here corresponds to the “R2” derivative referenced elsewhere. Viability data is expressed as a percentage of DMSO-treated cells. SEM is of three independent experiments and indicated by error bars. (**D**) Relative viability of parental or ABT-199-resistant OCI-AML2 cell lines transduced with a short hairpin targeting MCL-1 (MCL1-hp3, shown in panel **B**) at three viral doses. The resistant OCI-AML2 derivative used here corresponds to the “R2” derivative referenced elsewhere. Viability is shown relative to respective parental and resistant OCI-AML2 cell lines transduced with shGFP. Data are means ± SD from three experiments. ***p < 0.001 by Student’s t-test. (**E**) Time-to-resistance model of parental THP-1 cells treated with all possible one, two, and three-body combinations of DMSO/ABT-199/WEHI-539/shMCL-1. Lines are color-coded by the number of anti-apoptotic proteins targeted: black (zero), yellow (one), blue (two), red (three). One million cells were seeded at week zero and counted weekly for eight weeks. Cell counts in excess of one million were tabulated but a maximum of one million cells was replated each week. A running sum of all viable cells was estimated by extrapolating the weekly growth rate to a virtual cell count. The final three-target combination was run in replicate (red lines). All other conditions were single experiments. (**F**) Immunoblot showing MCL-1 levels immediately after hairpin knockdown of MCL-1 in THP-1 and following emergence of a resistant clone. Blots are representative of three replicate experiments. “shMCL1-3” sample was collected three days after puromycin selection. “shMCL1-3 + 199 + 539 reemergence” sample was collected at week eight from the resistant three-target combination cell population (red circle).

We have established that upregulation of MCL-1 and/or BCL-X_L_ is coincident with the acquisition of resistance to ABT-199 in AML cells. Using a combination of WEHI-539, which specifically targets BCL-X_L_^13^, and shRNA knockdown of MCL-1, we were able to completely resensitize ABT-199-resistant OCI-AML2 and THP-1 cell lines to ABT-199 (Figs 4A,B and S6), implicating the dynamic upregulation of MCL-1 and BCL-X_L_ as the driving force behind evolved resistance. Given their shift in anti-apoptotic dependence from BCL-2 to BCL-X_L_ and MCL-1, we reasoned that the evolved ABT-199-resistant cells should also be more susceptible to singular inhibition of BCL-X_L_ or MCL-1. To test this, ABT-199-resistant OCI-AML2 cells were subject to single treatment with WEHI-539 (Fig. 4C) or a short hairpin targeting MCL-1 (Fig. 4D), revealing an increased sensitivity to singly-targeted treatment compared to the paired parental line.[Table t1]

Lastly, we posited that simultaneous inhibition of multiple anti-apoptotic proteins at treatment outset might undermine the cells’ ability to acquire resistance to ABT-199 altogether. To test this idea, we used a long-term, time-to-resistance model in which parental THP-1 cells are treated with combinations of ABT-199, WEHI-539, and shRNA-based MCL-1 knockdown and tracked over the course of eight weeks (Figs 4E and S7)^14^. Targeting either BCL-X_L_ or MCL-1 in conjunction with ABT-199 treatment delayed the acquisition of drug resistance by 2–4 weeks. Targeting both BCL-X_L_ and MCL-1 pushed back the onset of resistance to a total of at least 7 weeks. Interestingly, in the three-target combination, western blot analysis of the resistant population indicated close to parental levels of MCL-1 (Fig. 4F), suggesting that the observed resistance may be merely the result of incomplete MCL-1 knockdown in a subset of cells that subsequently grew out over the course of several weeks. This idea is corroborated by the observation that a replicate line subject to the same hairpin and drug treatment failed to reemerge through 8 weeks of culture. In sum, we found no evidence that resistance to ABT-199 can be generated in the presence of combined BCL-X_L_ and MCL-1 inhibition.

## Discussion

The appeal of targeted therapies lies in the promise that, by exploiting a specific molecular weakness, a rationally designed therapy can kill cancer cells potently and selectively. Yet, in order for rational therapies to yield durable clinical responses, they must be paired with additional drugs capable of suppressing the cellular escape mechanisms that permit the acquisition of drug resistance. In most cases, these mechanisms are poorly defined, making acquired resistance difficult to plan for. Here, we sought to understand how AML cells acquire resistance to the selective BCL-2 inhibitor ABT-199. Prior studies have already demonstrated that ABT-199 treatment is capable of inducing apoptosis in AML cell lines, both in primary AML cells and in murine xenograft models of AML^1^. The work presented here is directed at understanding how AML cells might acquire resistance to the BH3 mimetic ABT-199 and how knowledge of those resistance mechanisms might be leveraged to design strategies to counteract drug resistance or preclude the development of resistance altogether.

Using ABT-199-resistant AML cell lines derived through chronic drug exposure as a model for acquired resistance, we identified MCL-1 and BCL-X_L_ as key mediators of resistance, substantiating earlier findings from our pathway-activating genetic screen. Western blot analysis of multiple evolved resistant cell lines revealed consistent upregulation of MCL-1 and/or BCL-X_L_ relative to their parental counterparts. Notably, this was even observed in the intrinsically resistant OCI-AML3 cell line, which expresses substantial levels of MCL-1 at baseline^2^, yet further increased its expression to drive resistance following chronic drug exposure. These observations suggest that the ability to upregulate MCL-1 and/or BCL-X_L_ in response to inhibition of BCL-2 is shared amongst AML cells, irrespective of their levels of baseline sensitivity to ABT-199. While the specific mechanisms mediating this upregulation remain unclear, our data indicate that the changes in overall MCL-1 and BCL-X_L_ protein levels are at least partially driven by stable upregulation of MCL-1 and BCL-X_L_ transcript levels and, in the case of MCL-1, by an increase in protein stability. These findings were made in cells cultured to drug resistance over the course of weeks. Acutely, we observed no change in BCL-X_L_ over a 48 hour drug exposure but did note a rapid increase in total MCL-1 protein content within two hours. However, similar to previous reports in chronic lymphocytic leukemia^15^, we were unable to detect a concomitant increase in MCL-1 transcript level within that time frame, potentially implicating increased protein stability. Importantly, these data suggest that the aggregate shift in cellular anti-apoptotic dependency from BCL-2 to MCL-1 and/or BCL-X_L_ that we report is likely comprised of acute and chronic components. Abrupt stabilization of MCL-1 in the near term may be followed by sustained transcriptional upregulation of MCL-1 and BCL-X_L_, or by gradual selection of high expressers through the process of acquiring resistance.

The coordinated upregulation of MCL-1 and/or BCL-X_L_ across many cell lines in response to chronic drug exposure implied a causal role in the acquisition of resistance to ABT-199. However, it remained possible that those changes were merely correlative and not directly related to the resistance phenotype. We addressed this possibility by BH3 profiling parental and ABT-199-resistant AML cells, which provided a functional readout of apoptotic disposition. BH3 profiling revealed a consistent increase in mitochondrial depolarization induced in the resistant cells by NOXA and HRK, suggesting a newfound reliance on their cognate anti-apoptotic binding partners MCL-1 and BCL-X_L_, respectively. These data demonstrate that the observed resistance to ABT-199 is driven by changes at the level of the mitochondria and is the direct result of increased anti-apoptotic reserve.

While our data points to upregulation of MCL-1 and BCL-X_L_ as the clear driving force behind acquired resistance to ABT-199, differential regulation of other BCL-2 family proteins could also play a role in mediating this resistance. For instance, our screens demonstrated that overexpression of BCL-2 is sufficient to confer resistance to ABT-199, likely by increasing the concentration of ABT-199 needed to fully inhibit BCL-2. However, BCL-2 was not observed to be upregulated in our resistant cell lines and indeed appeared to be downregulated in multiple resistant derivatives, making it an unlikely cause of acquired resistance. Finally, although the anti-apoptotic proteins BCL-w and BFL-1 were not queried here, our ability to fully resensitize resistant cells to ABT-199 by targeting MCL-1 and BCL-X_L_ suggests a negligible contribution.

Our data also implicates the presence of separate, incompletely understood processes that may underlie the upregulation of MCL-1 and BCL-X_L_. For instance, while we noted no change in expression of the pro-apoptotic protein BID and the pro-death effector protein BAX between parental and resistant cell lines, we did notice a modest decrease in BIM and a relative increase in BAK (Fig. S8). This observation, in light of BAK’s preference for BID over BIM^16^, may explain the increased depolarization induced by BID1 in resistant versus parental cells (Figs 2A,C and S3). However, upregulation of BAK, a terminal pro-apoptotic protein that is upregulated at both the mRNA (Fig. S9) and protein levels in ABT-199-resistant cells, is counterintuitive and suggests a paradoxical role for BAK in resistance to ABT-199. Consistent with this potential role in resistance, BAK knockdown partially resensitizes ABT-199-resistant THP-1 cells to ABT-199 (Fig. S10), suggesting a nonzero contribution to acquired resistance. Together, these findings suggest that while MCL-1 and BCL-X_L_ play dominant roles in driving the resistant state, pro-apoptotic proteins like BAK may also contribute in counterintuitive ways that merit further study.

Rational combination therapies are designed to preempt the anticipated mechanisms of resistance to the drug, often by preventing reactivation/downstream activation of the primary pathway or through proactive inhibition of a parallel pathway. For instance, reactivation of the MAPK pathway through multiple mechanisms drives resistance to BRAF inhibition and can be partially prevented with simultaneous inhibition of its downstream target MEK^17^,^18^,^19^,^20^. Similarly, certain *PIK3CA*/*KRAS*-mutant cancers, when treated with a MEK inhibitor, can activate collateral signaling through the PI3K pathway, which can be overcome by simultaneously administering a PI3K inhibitor^21^,^22^. Ideally, resistance could be targeted by focusing therapeutic attention on common, terminal nodes of resistance. Accordingly, it is worth underscoring that in each of our independently-evolved ABT-199-resistant AML lines, acquired resistance was accompanied by upregulation of MCL-1 and/or BCL-X_L_—anti-apoptotic BCL-2 family proteins not targeted by ABT-199. Because ABT-199 induces cell death by inhibiting a terminal negative regulator of apoptosis, perhaps it is not surprising that the mechanisms of acquired resistance also converge on terminal negative regulators of apoptosis. Moreover, this paradigm could be translationally important because it suggests that, despite the varied upstream pathways that may be responsible for dictating expression of anti-apoptotic BCL-2 family proteins, resistance to ABT-199 can always be overcome or preempted by targeting these key nodes at the level of the mitochondria. To that effect, we showed that acquired resistance to ABT-199 in AML cell lines can be reversed or entirely forestalled by simultaneously targeting BCL-2, MCL-1, and BCL-X_L_.

In sum, our data indicate that acquired resistance to ABT-199 in AML stems directly from a shift in cellular anti-apoptotic dependencies away from BCL-2 and toward MCL-1 and/or BCL-X_L_, as the cell struggles to maintain anti-apoptotic equipoise in the face of BCL-2 inhibition. Prior studies have identified in lymphoid malignancies a similar paradigm of resistance to ABT-199^9^, and in AML attendant mechanisms of resistance to the related compound ABT-737^2^,^23^. What had not been demonstrated until now was how AML cells adapt their anti-apoptotic profile to mitigate the effects of selective BCL-2 antagonism by ABT-199 and how that understanding might be exploited to reverse or proactively prevent drug resistance. Our findings are particularly notable in light of (1) promising new clinical trials data suggesting that ABT-199 is poised to have clinical impact for treatment of AML and (2) the ongoing development of selective, orally bioavailable inhibitors of BCL-X_L_^24^ and MCL-1^25^. Furthermore, recent work suggests that priming of BCL-X_L_-dependent cancer cells may provide a therapeutic window sufficient for on-target inhibition in cancer cells without affecting normal cells, allaying concerns about dose-dependent thrombocytopenia secondary to BCL-X_L_ inhibition^26^. It is also plausible that a full therapeutic effect could be achieved by creatively scheduling the administration of individual agents rather than delivering the full combination all at once. Clinically, combinatorial inhibition of anti-apoptotic BCL-2 family proteins may represent a viable strategy for resensitization of ABT-199-resistant neoplasms, offering recourse for patients that relapse on ABT-199 monotherapy. Alternatively, preemptive combination therapy could be administered as induction therapy, potentially enabling more durable initial remission by precluding the development of acquired resistance.

## Methods

### Cell lines and reagents

All cell lines were cultured at 37 °C in 5% CO_2_ and grown in RPMI 1640 with 10% fetal bovine serum (FBS) and 1% penicillin/streptomycin. OCI-AML2, NOMO-1, and OCI-AML3 cell lines were generously gifted by Anthony Letai (Dana Farber Cancer Institute). THP-1 and HL-60 cell lines were purchased from Duke University Cell Culture Facility (CCF). ABT-199-resistant and control cells were grown in media described above supplemented with [0.5 μM to 2.5 μM] ABT-199 or DMSO, respectively. Drugs were purchased from Selleck chemicals and were used at the following doses: 3 μM and 5 μM for ABT-199 (apoptosis assays), 1 μM for WEHI-539 (background dose for GI_50_ assay).

### *In vitro* adaptation of ABT-199-resistant AML cell lines

Cell lines resistant to ABT-199 were generated through chronic drug exposure as previously described^10^. In short, 4E6 parental cells were plated in a 15 cm dish and treated with a starting dose of ABT-199 equivalent to the GI_50_ of the cell line. A second plate of parental cells was simultaneously plated with an equivalent quantity of DMSO as a paired control. Cells in both dishes were subsequently observed and counted weekly in parallel. For cells cultured in drug, ABT-199 doses were increased in increments of 500 nM as soon as the cell population stabilized. Cell lines were considered fully resistant when they could maintain their population in media containing 2.5 μM ABT-199.

### Preparation of lentivirus for pathway activating screen and shRNA MCL-1 knockdown

Lentivirus particles were produced through transient transfection of 293 T cells using a three-plasmid system: expression clone + VSVG + δVPR as previously described^10^.

### Pathway-activating screen

We infected discrete populations of OCI-AML2 and MOLM13 cells with lentivirus encoding the expression of each of 39 individual constitutive activator constructs, each driven by a moderate PGK promoter, from a previously described cDNA library^10^. Lentiviruses were produced and applied as above. Infected cells were subject to three days of puromycin selection prior to seeding into 96-well plates for GI_50_ assay, described below. Candidate genes/pathways that shifted the GI_50_ of both respective cell lines to at least 1 μM were selected as candidates for followup.

Pathway activating constructs were previously cloned and sequence verified by members of our lab^10^; all constructs used were also publically available (Addgene plasmid #64602-64649). pMSCV-puro-mMcl-1 was a gift from Joseph Opferman (Addgene plasmid #32980). Human MCL-1 ORF was purchased at GeneCopoeia (product ID: Y4182).

### BH3 profiling

OCI-AML2, THP-1, NOMO-1, OCI-AML3 cells were BH3-profiled as previously described^27^. All peptides were used at a concentration of 100 μM, unless otherwise indicated.

### GI_50_ assay

Cells were seeded in 96-well plates at 5000 cells per well. After 24 hours, cells were treated, by row, with a 10-fold serial dilution of indicated drug in DMSO to yield final drug concentrations of 20, 2, 0.2, 0.02, 0.002, 0.0002, 0.00002, and 0.000002 μM. A final well was treated with only DMSO. CellTiter-Glo luminescent viability assay (Promega) was used to measure cell viability 72 hours after addition of drug. Each treatment condition was represented by three individual experiments. Relative viability was calculated by normalizing raw luminescence counts to DMSO-treated wells. For experiments involving two drugs, a second background drug was kept at a constant concentration across all wells except for the DMSO control. Viability in two-drug experiments was normalized to luminescence of secondary drug-only well. For pathway-activating screen, GI_50_ values were calculated by fitting each individual experiment to a 4-parameter logistic curve using GraphPad/Prism 6 software and selecting the dose at which cell viability equals 50% of DMSO-treated viability.

### Apoptosis assay

250,000 cells were seeded into each well of a six-well plate and treated with indicated quantity of drug or DMSO. Cells were incubated for 48 hours, washed twice with phosphate-buffered saline (PBS), and resuspended in Annexin V binding buffer (10 mM Hepes, 140 mM NaCl, 2.5 mM CaCl_2_; BD Biosciences). Phosphatidylserine externalization was measured using APC (allophycocyanin)-conjugated Annexin V (BD Biosciences). 7-AAD (BD Biosciences) was used as the viability probe. Experiments were analyzed at 20,000 counts per sample using BD FACSVantage SE. Gating strategy was defined using stained/unstained cells.

### Quantitative Reverse Transcription PCR

RNA extraction, cDNA synthesis and quantitative real-time PCR was performed as previously described^10^. The following primers were used: human GAPDH, 5′-CCCACTCCTCCACCTTTGAC-3′ (forward) and 5′-ACCCTGTTGCTGTAGCCAAA-3′ (reverse); human MCL-1, 5′-GGACAAAACGGGACTGGCTA-3′ (forward) and 5′-CAGCAGCACATTCCTGATGC-3′ (reverse); human BCL-X_L_, 5′-TGACCACCTAGAGCCTTGGA-3′ (forward) and 5′-CAGTCATGCCCGTCAGGAAC-3′ (reverse). Average cycle thresholds (C_t_) were calculated for each gene normalized to the reference gene GAPDH. Relative gene expression was determined using the ΔΔC_t_ method.

qRT-PCR data was compiled as means and standard deviations. For OCI-AML2, differences in MCL-1 and BCL-X_L_ expression between parental and all three derivatives were detected using one-way ANOVA. Subsequently, Dunnett’s multiple comparisons test was applied to evaluate significant differences in expression for each resistant derivative relative to parental control. For NOMO-1 and THP-1, differences between means of MCL-1 or BCL-X_L_ expression in resistant relative to parental cell lines was examined using Student’s t-test.

### Western blotting and antibodies

Immunoblotting was performed as previously described^10^. Membranes were probed with primary antibodies recognizing MCL-1, BCL-X_L_, Bcl-2, BIM, BID, BAX, BAK, BAD p-S112, BAD p-S136, total BAD at a 1:1000 dilution and β-actin at 1:5000. Secondary goat anti-rabbit IgG-HRP was applied at 1:5000. All primary antibodies were purchased from Cell Signaling Technology; secondary antibodies were purchased from Santa Cruz Biotechnology.

### shRNA constructs

TRC shRNA clones were acquired from the Duke RNAi Facility as glycerol stocks. Constructs were prepared as lentivirus and used for viral transduction as described above.

## Additional Information

**How to cite this article**: Lin, K. H. *et al*. Targeting MCL-1/BCL-X_L_ Forestalls the Acquisition of Resistance to ABT-199 in Acute Myeloid Leukemia. *Sci. Rep.* **6**, 27696; doi: 10.1038/srep27696 (2016).

## Supplementary Material

Supplementary Information

## Figures and Tables

**Table 1 t1:** shRNA Constructs Used to Knockdown MCL-1.

**Construct**	**TRC ID**	**Sequence**
shMCL-1 (1)	TRCN0000005517	GCTAAACACTTGAAGACCATA
shMCL-1 (2)	TRCN0000197024	GAGCTGGTTTGGCATATCTAA
shMCL-1 (3)	TRCN0000196390	GCCTAGTTTATCACCAATAAT

TRC; The RNAi Consortium.

## References

[b1] Pan R. . Selective BCL-2 inhibition by ABT-199 causes on-target cell death in acute myeloid leukemia. Cancer Discov. 4, 362–375, doi: 10.1158/2159-8290.CD-13-0609 (2014). 24346116 PMC3975047

[b2] Konopleva M. . Mechanisms of apoptosis sensitivity and resistance to the BH3 mimetic ABT-737 in acute myeloid leukemia. Cancer Cell. 10, 375–388, doi: 10.1016/j.ccr.2006.10.006 (2006). 17097560

[b3] Ni Chonghaile T. & Letai A. Mimicking the BH3 domain to kill cancer cells. Oncogene. 27 Suppl 1, S149–157, doi: 10.1038/onc.2009.52 (2008). 19641500 PMC3733265

[b4] Roberts A. W. . Substantial susceptibility of chronic lymphocytic leukemia to BCL2 inhibition: results of a phase I study of navitoclax in patients with relapsed or refractory disease. J Clin Oncol. 30, 488–496, doi: 10.1200/JCO.2011.34.7898 (2012). 22184378 PMC4979082

[b5] Souers A. J. . ABT-199, a potent and selective BCL-2 inhibitor, achieves antitumor activity while sparing platelets. Nat Med. 19, 202–208, doi: 10.1038/nm.3048 (2013). 23291630

[b6] Konopleva M. . A Phase 2 Study of ABT-199 (GDC-0199) in Patients with Acute Myelogenous Leukemia (AML). Presented at: *56th ASH Annual Meeting and exposition;* San Francisco, CA. December **6–9** 2014; (Abstract 118).

[b7] Yecies D., Carlson N. E., Deng J. & Letai A. Acquired resistance to ABT-737 in lymphoma cells that up-regulate MCL-1 and BFL-1. Blood. 115, 3304–3313, doi: 10.1182/blood-2009-07-233304 (2010). 20197552 PMC2858493

[b8] Vogler M. . Concurrent up-regulation of BCL-XL and BCL2A1 induces approximately 1000-fold resistance to ABT-737 in chronic lymphocytic leukemia. Blood. 113, 4403–4413, doi: 10.1182/blood-2008-08-173310 (2009). 19008458

[b9] Choudhary G. S. . MCL-1 and BCL-XL-dependent resistance to the BCL-2 inhibitor ABT-199 can be overcome by preventing PI3K/AKT/mTOR activation in lymphoid malignancies. Cell Death Dis. 6, e1593, doi: 10.1038/cddis.2014.525 (2015). 25590803 PMC4669737

[b10] Martz C. A. . Systematic identification of signaling pathways with potential to confer anticancer drug resistance. Science Signaling. 7, doi: 10.1126/scisignal.aaa1877 (2014). PMC4353587 25538079

[b11] Deng J. . BH3 profiling identifies three distinct classes of apoptotic blocks to predict response to ABT-737 and conventional chemotherapeutic agents. Cancer Cell. 12, 171–185, doi: 10.1016/j.ccr.2007.07.001 (2007). 17692808

[b12] Ryan J. & Letai A. BH3 profiling in whole cells by fluorimeter or FACS. Methods. 61, 156–164, doi: 10.1016/j.ymeth.2013.04.006 (2013). 23607990 PMC3686919

[b13] Lessene G. . Structure-guided design of a selective BCL-X(L) inhibitor. Nat Chem Biol. 9, 390–397, doi: 10.1038/nchembio.1246 (2013). 23603658

[b14] Misale S. . Vertical suppression of the EGFR pathway prevents onset of resistance in colorectal cancers. Nature Communications. 6, doi: 10.1038/ncomms9305 (2015). PMC4595628 26392303

[b15] Mazumder S., Choudhary G. S., Al-Harbi S. & Almasan A. Mcl-1 phosphorylation defines ABT-737 resistance that can be overcome by increased NOXA expression in leukemic B-cells. Cancer Research. 72, 3069–3079, doi: 10.1158/0008-5472.CAN-11-4106 (2012). 22525702 PMC3377792

[b16] Sarosiek K. A. . BID preferentially activates BAK while BIM preferentially activates BAX, affecting chemotherapy response. Mol Cell. 51, 751–765, doi: 10.1016/j.molcel.2013.08.048 (2013). 24074954 PMC4164233

[b17] Long G. V. . Combined BRAF and MEK inhibition versus BRAF inhibition alone in melanoma. N Engl J Med. 371, 1877–1888, doi: 10.1056/NEJMoa1406037 (2014). 25265492

[b18] Flaherty K. T. . Combined BRAF and MEK inhibition in melanoma with BRAF V600 mutations. N Engl J Med. 367, 1694–1703, doi: 10.1056/NEJMoa1210093 (2012). 23020132 PMC3549295

[b19] Heidorn S. J. . Kinase-dead BRAF and oncogenic RAS cooperate to drive tumor progression through CRAF. Cell. 140, 209–221, doi: 10.1016/j.cell.2009.12.040 (2010). 20141835 PMC2872605

[b20] Poulikakos P. I., Zhang C., Bollag G., Shokat K. M. & Rosen N. RAF inhibitors transactivate RAF dimers and ERK signalling in cells with wild-type BRAF. Nature. 464, 427–430, doi: 10.1038/nature08902 (2010). 20179705 PMC3178447

[b21] Wee S. . PI3K pathway activation mediates resistance to MEK inhibitors in KRAS mutant cancers. Cancer Res. 69, 4286–4293, doi: 10.1158/0008-5472.CAN-08-4765 (2009). 19401449

[b22] Engelman J. A. . Effective use of PI3K and MEK inhibitors to treat mutant Kras G12D and PIK3CA H1047R murine lung cancers. Nat Med. 14, 1351–1356, doi: 10.1038/nm.1890 (2008). 19029981 PMC2683415

[b23] Pan R. . Inhibition of Mcl-1 with the pan–Bcl-2 family inhibitor (–)BI97D6 overcomes ABT-737 resistance in acute myeloid leukemia. Blood, doi: 10.1182/blood-2014-10-604975 (2015). PMC4504949 26045609

[b24] Tao Z. F. . Discovery of a Potent and Selective BCL-XL Inhibitor with *in vivo* Activity. ACS Med Chem Lett. 5, 1088–1093, doi: 10.1021/ml5001867 (2014). 25313317 PMC4190639

[b25] Leverson J. D. . Potent and selective small-molecule MCL-1 inhibitors demonstrate on-target cancer cell killing activity as single agents and in combination with ABT-263 (navitoclax). Cell Death Dis. 6, e1590, doi: 10.1038/cddis.2014.561 (2015). 25590800 PMC4669759

[b26] Leverson J. D. . Exploiting selective BCL-2 family inhibitors to dissect cell survival dependencies and define improved strategies for cancer therapy. Science Translational Medicine. 7, doi: 10.1126/scitranslmed.aaa4642 (2015). 25787766

[b27] Winter P. . RAS signaling promotes resistance to JAK inhibitors by suppressing BAD-mediated apoptosis. Science Signaling. 7, doi: 10.1126/scisignal.2005301 (2014). PMC4353591 25538080

