# Supplementary material for: Targeting MCL-1/BCL-XL Forestalls the Acquisition of Resistance to ABT-199 in Acute Myeloid Leukemia
Source: Sci Rep. 2016 Jun 10;6:27696. doi: 10.1038/srep27696 (PMC4901329; doi:10.1038/srep27696)
Supplement: Supplementary Information [file srep27696-s1.pdf]

Targeting MCL-1/BCL-X_L Forestalls the Acquisition of Resistance to ABT-199 in Acute Myeloid Leukemia

Authors: Kevin H. Lin[1], Peter S. Winter[1,2], Abigail Xie[1], Cullen Roth[1,2], Colin A. Martz[1], Elizabeth M. Stein[1], Gray R. Anderson[1], Jennifer P. Tingley[1], and Kris C. Wood[1]*

Affiliations:
[1]Department of Pharmacology and Cancer Biology, Duke University, Durham, NC 27710, USA;
[2]Program in Genetics and Genomics, Duke University, Durham, NC 27710, USA

Figure S1

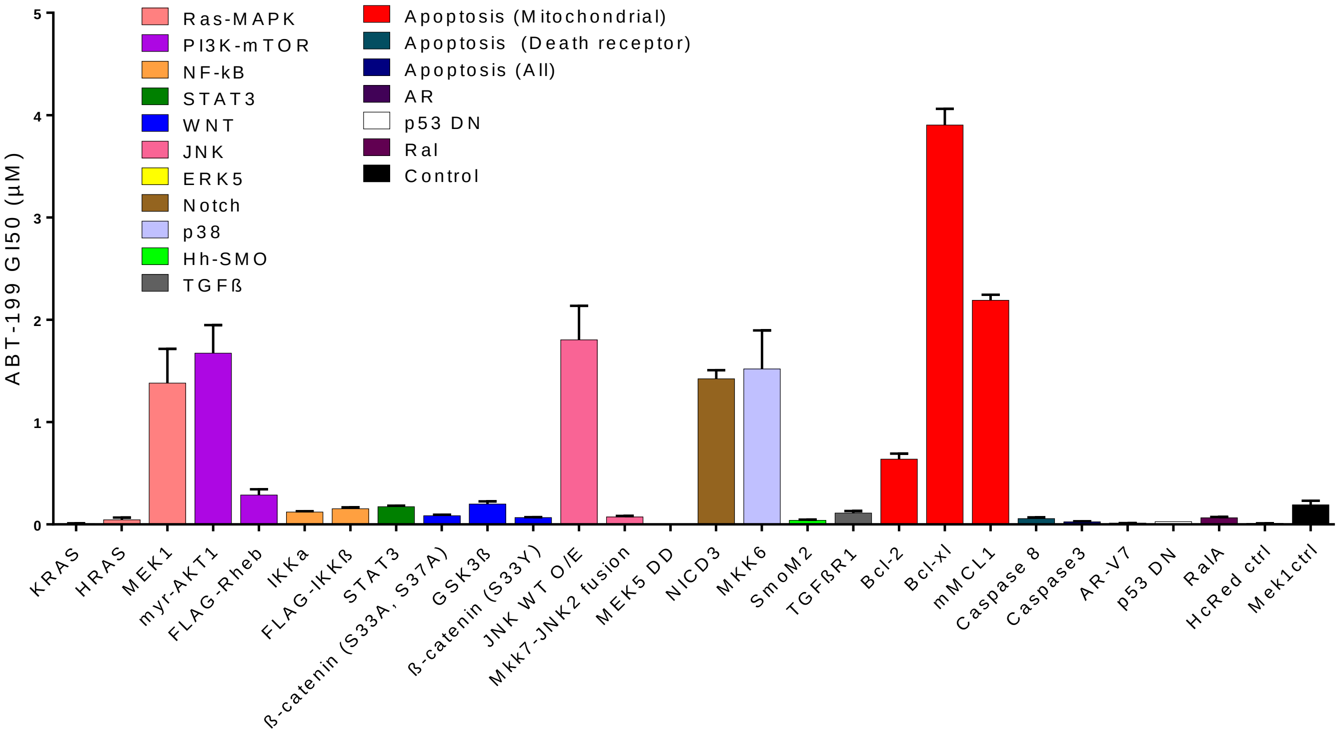

Supplementary Figure 1. Pathway activating screen in OCI-AML2 nominates BCL-X_L and MCL-1. Discrete populations of OCI-AML2 cells were individually transduced with indicated pathway-activating cDNA constructs. Drug sensitivity of each population was evaluated with a GI_{50} assay; data shown are mean GI_{50} (µM) ± SEM. Immunoblots showing overexpression of BCL-X_L and MCL-1 are shown in Figure 1.

Figure S2

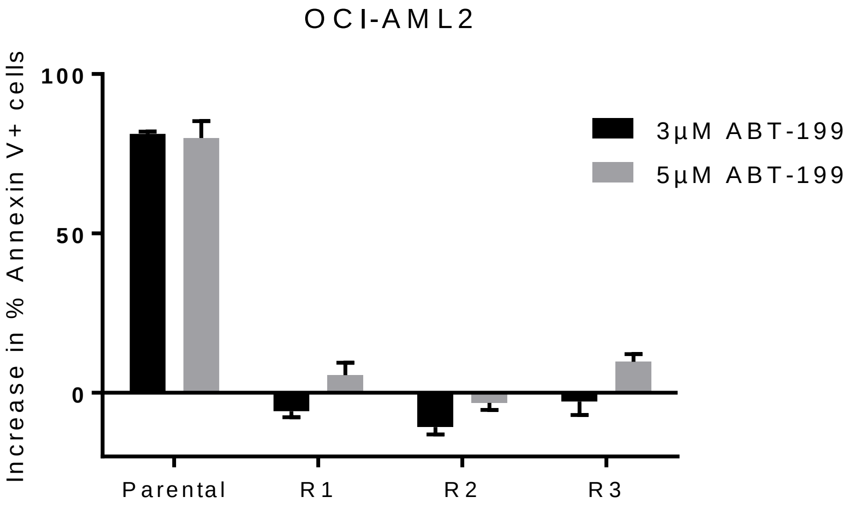

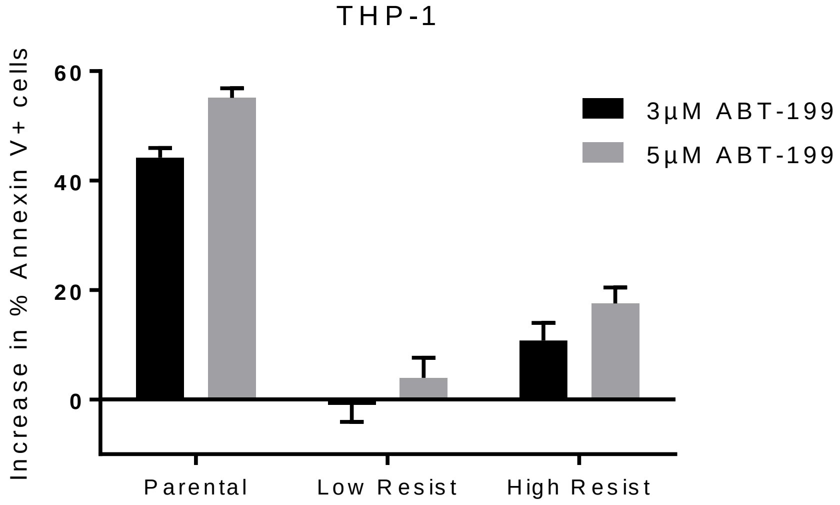

Supplementary Figure 2. Comparing drug-induced apoptosis in ABT-199-resistant versus parental AML cells. Increase in annexin V-positive OCI-AML2 or THP-1 cells after 48 hours treatment with 3µM or 5µM ABT-199. Data shown are mean increases in annexin V-positive cells relative to matched cells treated with DMSO ± SD from three independent experiments.

Figure S3

A

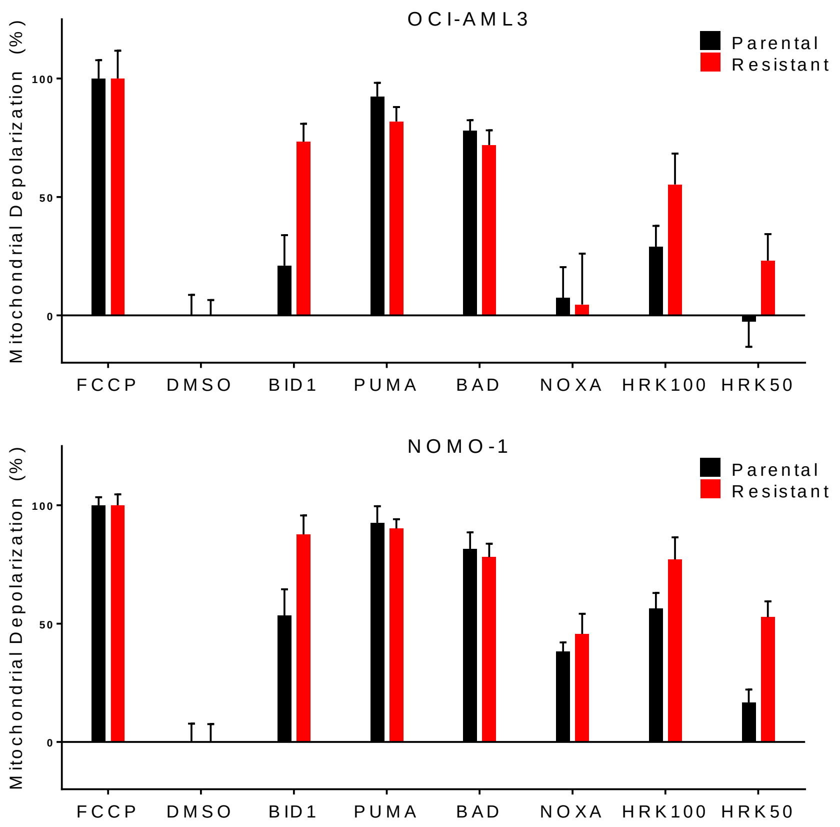

B

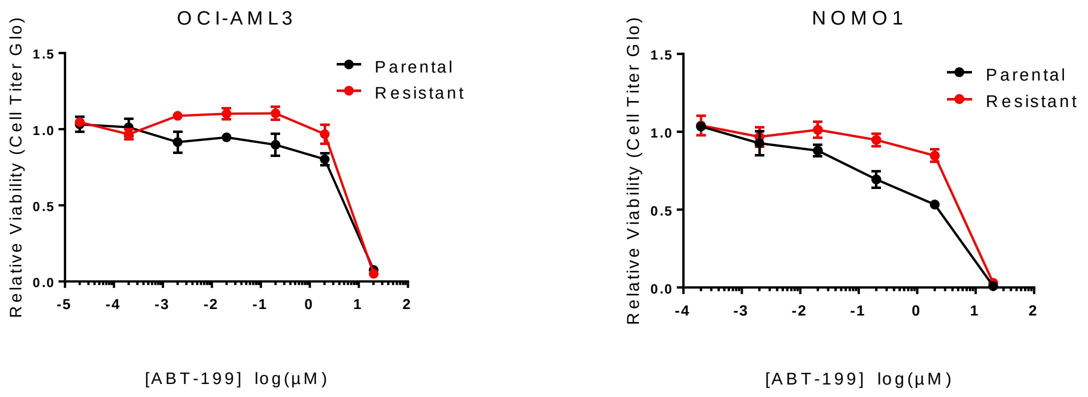

Supplementary Figure 3. Characterization of parental and resistant OCI-AML3 and NOMO-1. (A) BH3 profiling, as in Figure 2, of parental and resistant OCI-AML3 and NOMO-1 cells reveals increased mitochondrial depolarization in resistant lines in response to NOXA and HRK peptides. Viability data is expressed as a percentage of DMSO-treated cells. SEM is of three independent experiments and indicated by error bars. **(B)** ABT-199 dose-response curves for parental and resistant derivatives of OCI-AML3 and NOMO-1. Viability data is expressed as a percentage of DMSO-treated cells. SEM is of three independent experiments and indicated by error bars.

Figure S4

A

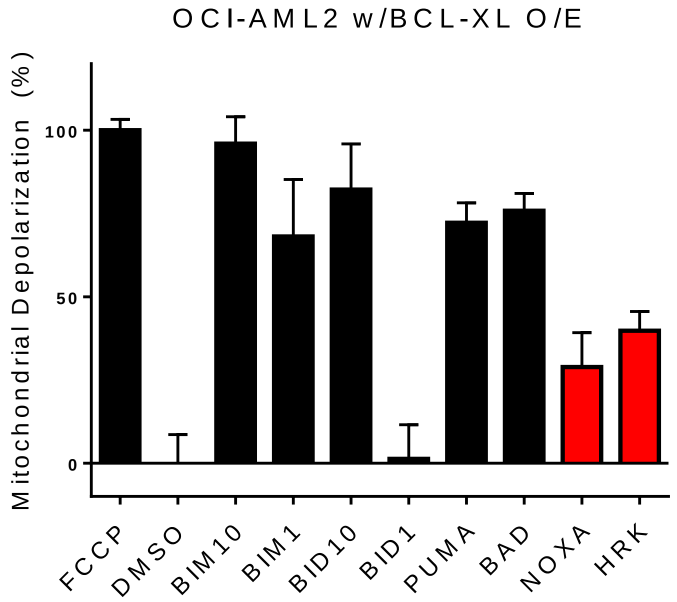

B

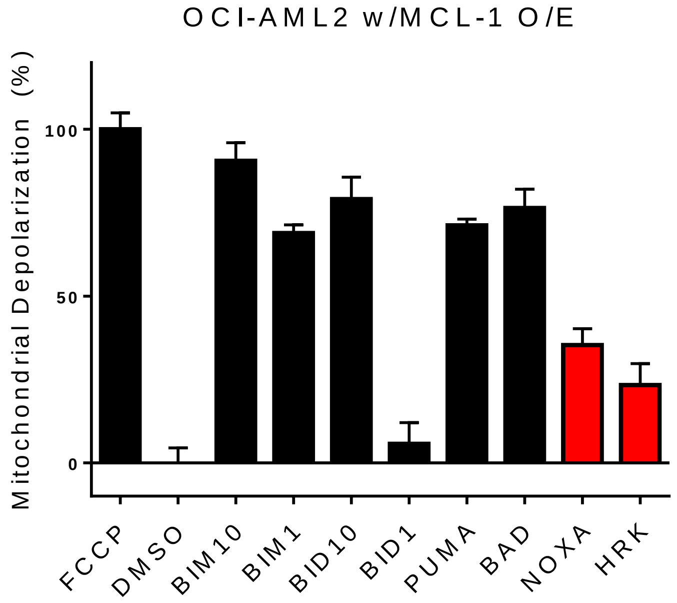

Supplementary Figure 4. BH3 profiling of OCI-AML2 cells overexpressing BCL-X_L or mMCL-1. BH3 profiling, as in Figure 2, was performed in parental OCI-AML2 cells transduced with indicated lentiviral construct. Artificial overexpression of **(A)** BCL-X_L and **(B)** MCL-1 in parental OCI-AML2 cells results in preferentially increased mitochondrial depolarization upon treatment with HRK and NOXA, respectively. Unless otherwise indicated, peptide concentration used was 100 µM. Data shown represent the mean ± SD of three independent experiments per peptide.

Figure S5

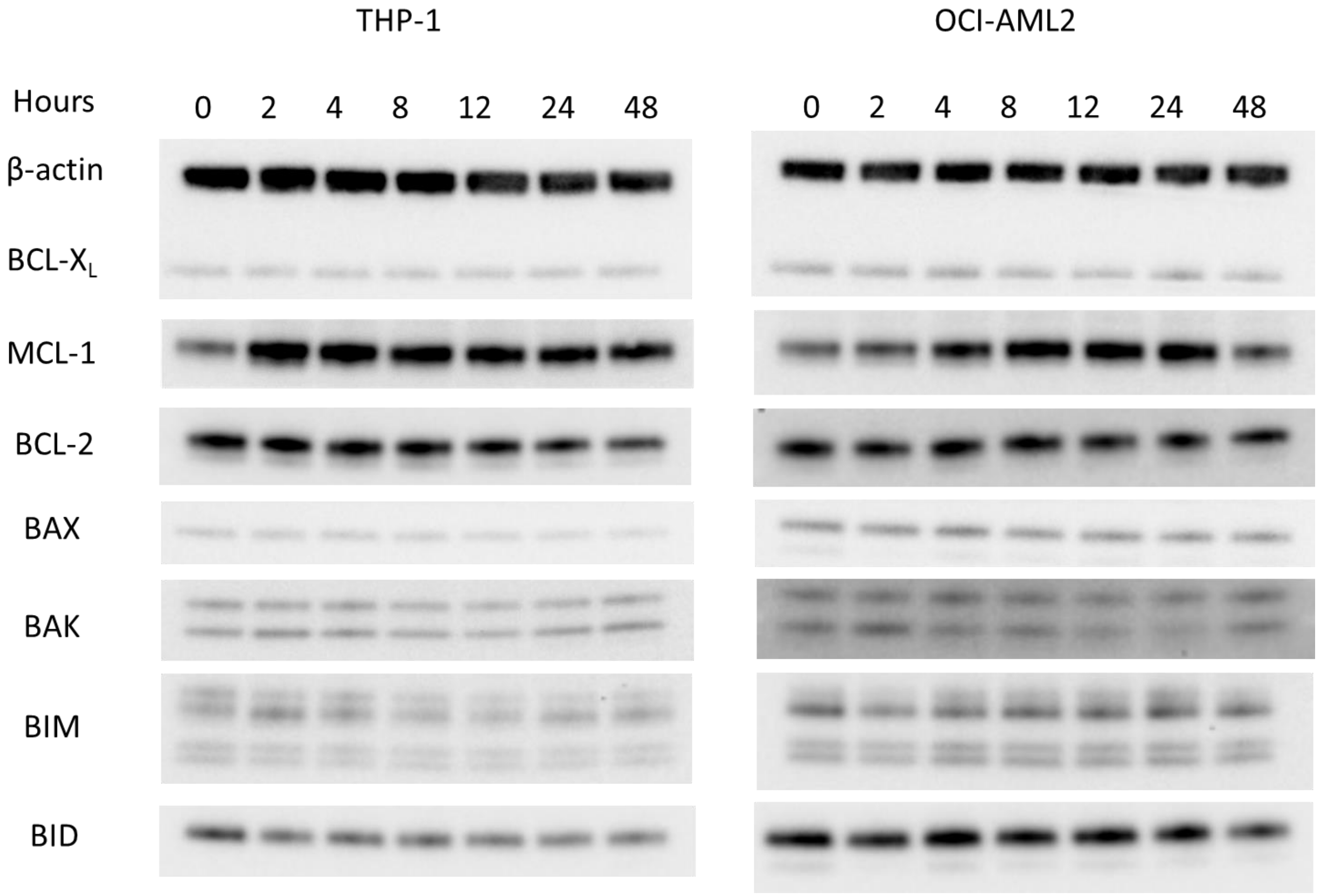

Supplementary Figure 5. Western blot analysis of BCL-2 family proteins. Western blot analysis of parental THP-1 and OCI-AML2 harvested at the indicated time points following treatment with 1µM ABT-199. Blots are representative of two replicate experiments.

Figure S6

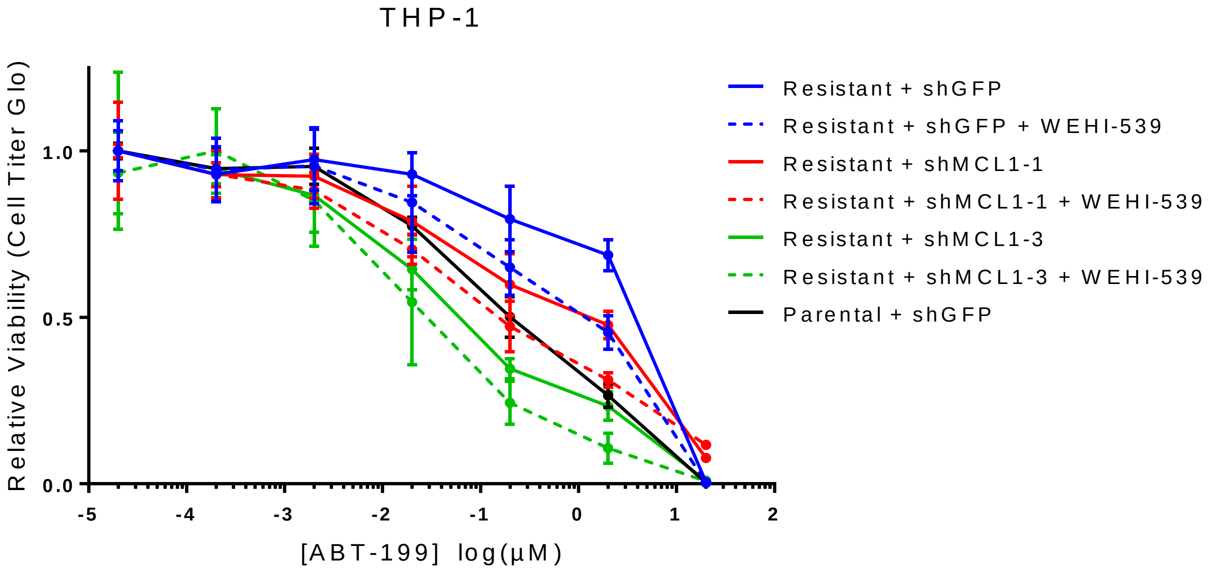

Supplementary Figure 6. Resensitization of high resistance THP-1 to ABT-199. ABT-199 dose-response curves for an ABT-199-resistant THP-1 line and subsequent resensitization of that line using the BCL-X_L inhibitor WEHI-539 and/or either of two independent hairpins targeting MCL-1. Viability data is expressed as a percentage of DMSO-treated cells. SEM is of three independent experiments and indicated by error bars.

Figure S7

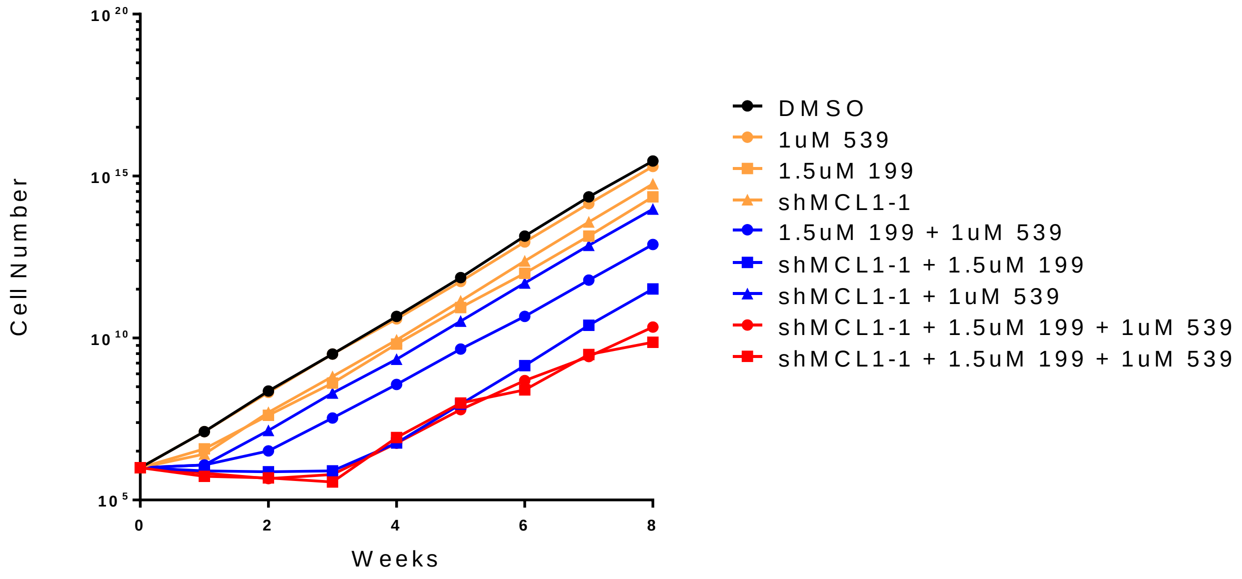

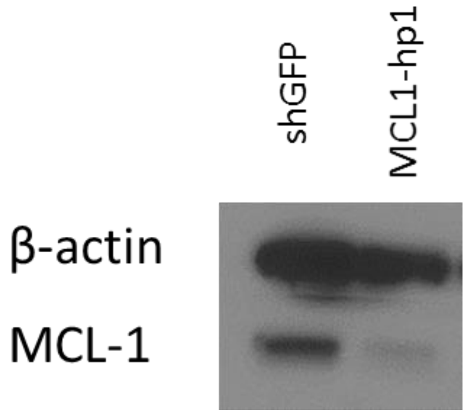

Supplementary Figure 7. Time-to-resistance model for THP-1 cells with MCL-1 hairpin 1 (partial knockdown). THP-1 cells were seeded at one million cells per dish, treated as indicated, and counted and replated weekly. Lines are color-coded by the number of anti-apoptotic proteins targeted: black (zero), yellow (one), blue (two), red (three). The final three-target combination was run in replicate. All other conditions were single experiments. The MCL-1 hairpin 1 used here provides incomplete knockdown. Corresponding immunoblot demonstrating hairpin knockdown in parental THP-1 is representative of three replicate experiments.

Figure S8

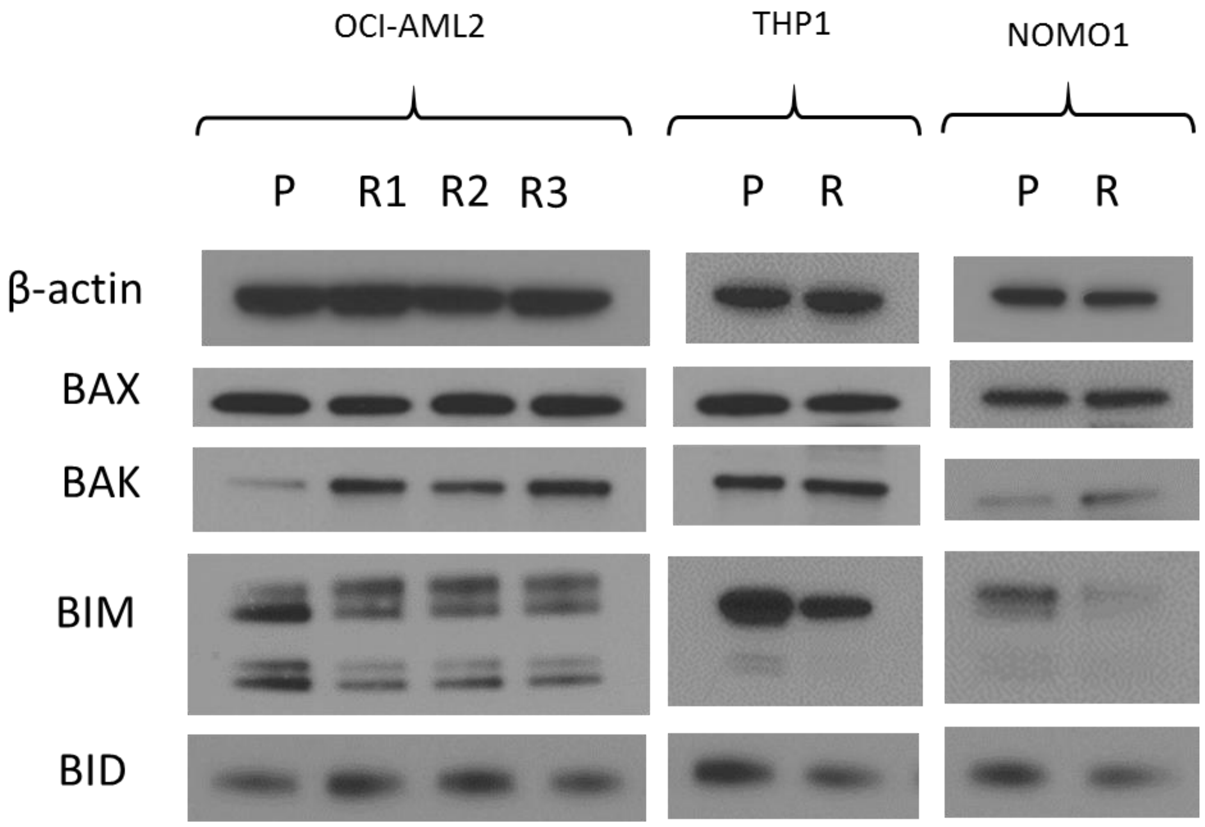

Supplementary Figure 8. Western blot analysis of BCL-2 family proapoptotic sensitizers and effectors. Western blot analysis of paired parental (P) and derived-resistant (R) cell lines immunoblotted as shown. Blots are representative of three replicate experiments.

Figure S9

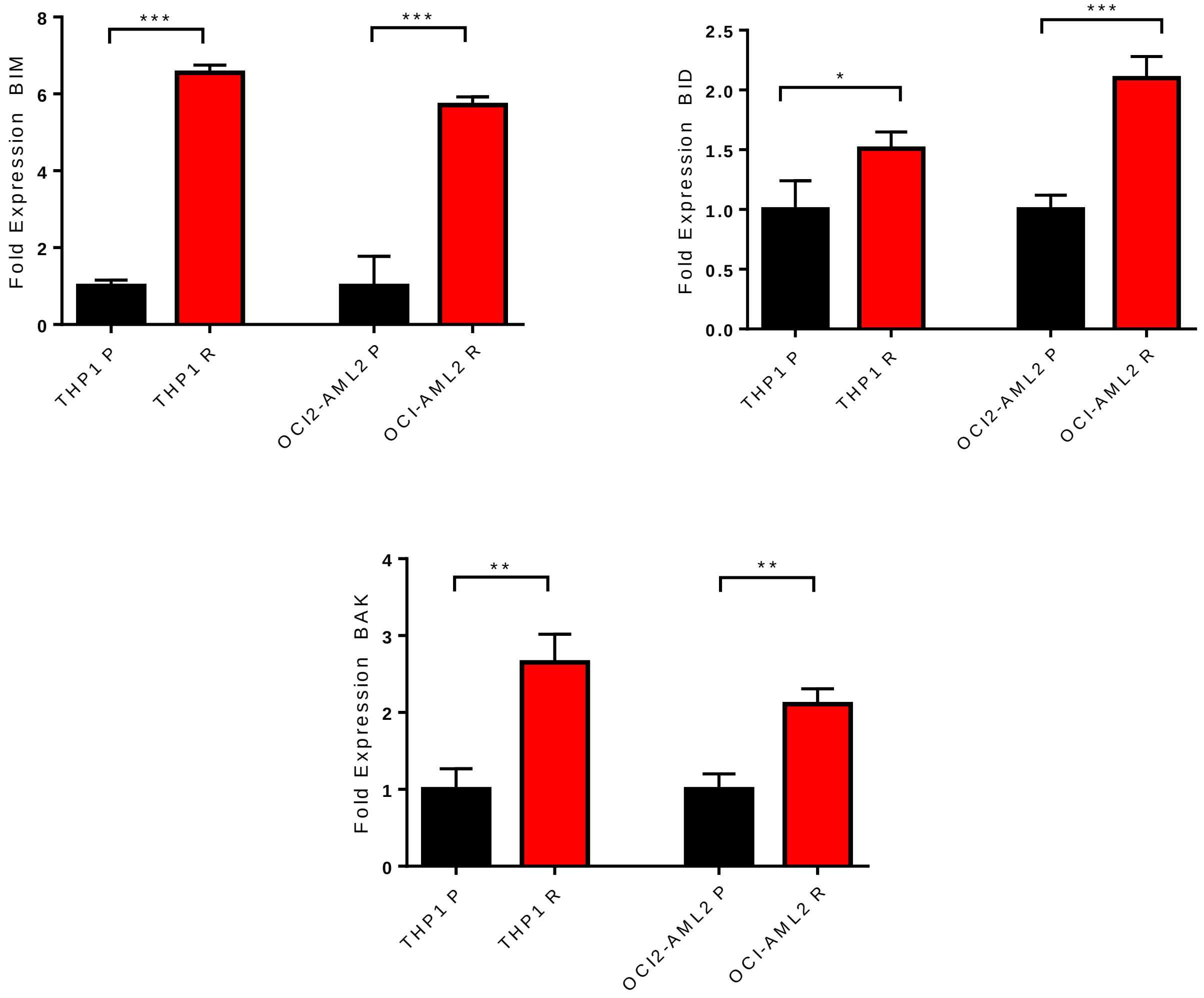

Supplementary Figure 9. ABT-199-resistant cells transcriptionally upregulate the pro-apoptotic proteins BIM, BID, BAK. qRT-PCR analysis of MCL-1 and BCL-X_L in parental (black bars) versus resistant derivatives (red bars) of OCI-AML2 and THP1 cell lines. The resistant OCI-AML2 derivative used here corresponds to the "R2" derivative referenced elsewhere. The resistant THP1 derivative used here corresponds to the "High Resistance" derivative referenced elsewhere. Data are means ± SD from three experiments. *p <0.05, ***p<0.001, ****p<0.0001 by Student's t-test.

Figure S10

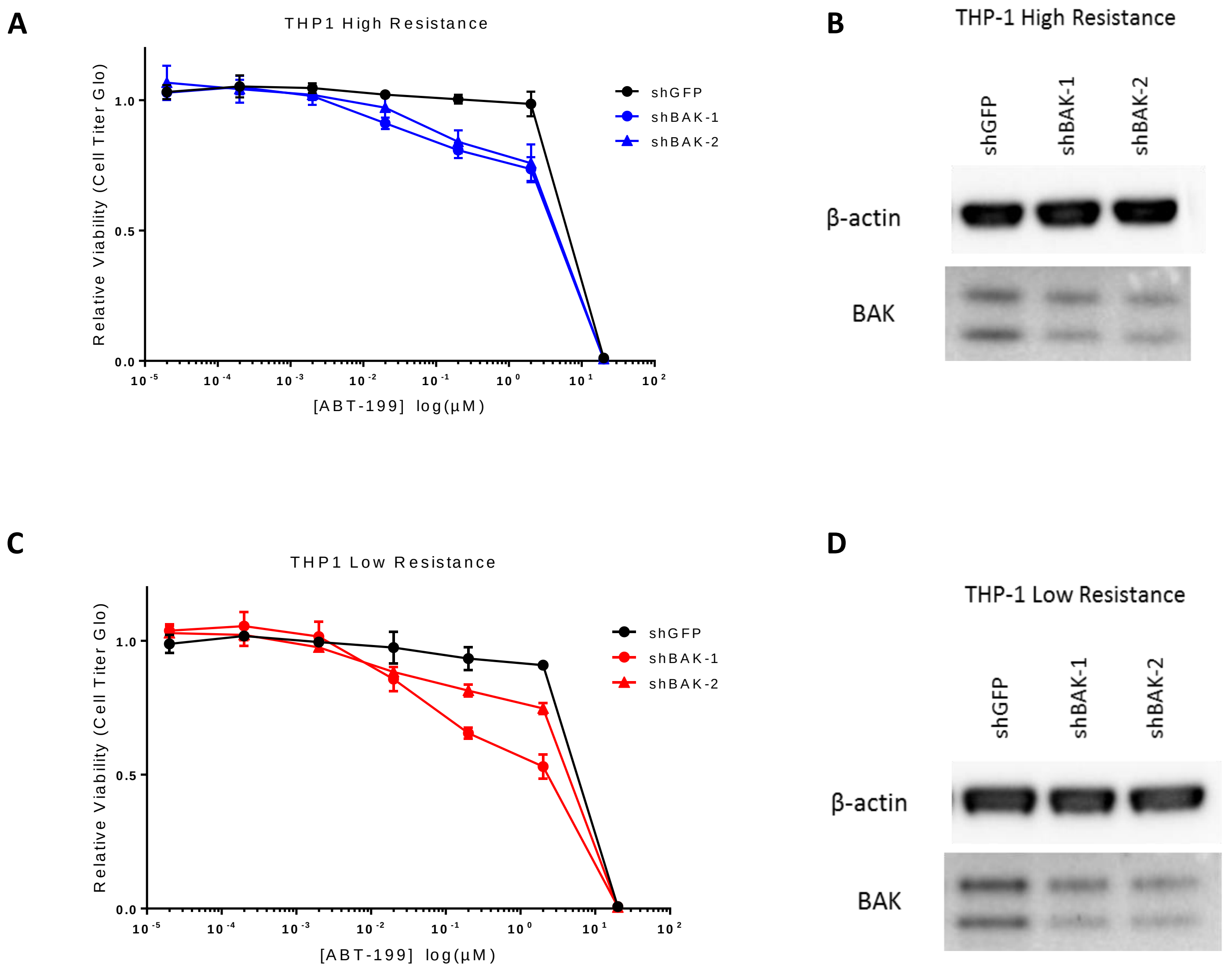

Supplementary Figure 10. BAK knockout in ABT-199-resistant THP-1 cell lines partially reverses resistance to ABT-199. (A)(C) ABT-199 dose-response curves for parental and differentially resistant derivatives of THP-1 transduced with short hairpins targeting either GFP or BAK. Viability data is expressed as a percentage of DMSO-treated cells. SEM is of three independent experiments and indicated by error bars. **(B)(D)** Western blot analysis of resistant THP-1 cells transduced with short hairpins targeting either shGFP or BAK. Blots are representative of two replicate experiments.

Table S1: List of pathway-activating constructs and controls used in this study.

Pathway	Construct	Official symbol and NM#	Functionally validated?	Functional Validation Method
Ras-MAPK	Kras (G12V)	KRAS NM_004985.3 - human variant b	Yes	Western (P-ERK)
	Hras (G12V)	HRAS NM_005343.2 - human variant 1	Yes	Western (P-ERK)
	MEK1 (S218D,S222D)	MAP2K1 NM_002755.3 - human	Yes (both +/- V5)	Western (P-ERK)
PI3K-AKT-mTOR	myr-FLAG-PIK3CA	PIK3CA NM_174574.1 - bovine	Yes	Western (P-AKT)
	myr-FLAG-AKT1	AKT1 NM_005163.2 - human variant 1	Yes	Western (P-AKT, P-S6K1)
	FLAG-Rheb (Q64L)	RHEB NM_005614.3 - human	Yes	Western (P-S6K1)
NF-κB	IKKα (S176E,S180E)	CHUK NM_001278.3 - human	Yes	Reporter (NF-κB_Luc)
	FLAG-IKKβ (S177E,S181E)	IKBKB NM_001556.2 - human variant 1	Yes	Reporter (NF-κB_Luc)
Jak/Stat	JAK2 (V617F)	JAK2 NM_004972.3 - human	Yes	Reporter (Stat_Luc)
	Stat3 (A662C,N664C,V667L)	STAT3 NM_139276.2 - human variant 1	Yes	Reporter (Stat_Luc)
Wnt/b-catenin	β-catenin (S33A, S37A, T41A, S45A)	Ctnnb1 NM_001165902.1 - mouse variant 2	Yes (both +/- V5)	Reporter (TCF-LEF_Luc)
	GSK3β (K85A)	GSK3B NM_001146156.1 - human variant 2	Yes	Reporter (TCF-LEF_Luc)
	β-catenin (S33Y)	CTNNB1 NM_001904.3 - human variant 1	Yes (both +/- V5)	Reporter (TCF-LEF_Luc)
JNK	JNK2 WT O/E (MAPK9)	MAPK9 NM_002752.4 - human variant JNK2-a2	No	Reporter (AP1_Luc)
	Mkk7-JNK2 fusion	Map2k7 NM_011944.3 linker MAPK9 NM_002752.4-mouse Mkk7 variant 2 plus human variant JNK2-a2	Yes	Reporter (AP1_Luc)
ERK5	MEK5 DD(S311D,T315D)	MAP2K5 NM_145160.2 - human variant 1	No	Western (ERK5 laddering)
	myr-FLAG -MEK5	MAP2K5 NM_145160.2 - human variant 1	Yes	Western (ERK5 laddering)
Notch	Notch1 intracellular domain	NOTCH1 NM_017617.3 - human, intracellular domain see sequence	Yes (both +/- V5)	Reporter (HES1_Luc)
	Notch3 intracellular domain	NOTCH3 NM_000435.2 - human ,intracellular domain see sequence	Yes (both +/- V5)	Reporter (HES1_Luc)
p38	p38 WT O/E (MAPK14)	MAPK14 NM_139012.2 - human variant 2	Yes	Western (P-p38)
	FLAG-MKK6 (S207E,T211E)	MAP2K6 NM_002758.3 - human	Yes	Western (P-p38)
Hedgehog	Gli2 truncation	GLI2 NM_005270.4 - human, truncation mutant see sequence	Yes	Reporter (Gli_Luc)
	SmoM2 (W535L)	SMO NM_005631.4 - human	Yes	Reporter (Gli_Luc)
TGFβ	TGFβR1	TGFBR1 NM_004612.2 - human variant 1	Yes	Immunofluorescence (P-Smad2/3)
Mitochondrial apoptosis (intrinsic)	BCL2	BCL2 NM_000633.2 - human variant alpha	Yes	Western (cleaved caspase 9)
	BCL-XL	BCL2L1 NM_138578.1 - human variant 1	Yes	Western (cleaved caspase 9)
Death receptor apoptosis (extrinsic)	Caspase-8 (C360A)	CASP8 NM_033355.3 - human variant B	Yes	Western (cleaved caspase 8)
All apoptosis	Caspase-3 (C163A)	CASP3 NM_032991.2 - human variant beta	Yes	Western (cleaved caspase 3/7)
Estrogen receptor	ERα (Y537S mutant)	ESR1 NM_000125.3 - human variant 1	Yes	Reporter (ERE_Luc)
Androgen receptor	AR-V7	AR NM_000044.3 - human variant 1,splice isoform see sequence	Yes (both +/- V5)	Western (ARE_Luc)
Hippo	FLAG-YAP2 (5SA)	YAP1 NM_001195044.1 - human variant 3	Yes	Immunofluorescence (nuclear YAP)
	Lats2 kinase dead (K697R)	LATS2 NM_014572.2 - human	Yes (both +/- V5)	Immunofluorescence (nuclear YAP)
p53	p53 (dominant negative R175H mutant)	TP53 NM_001126114.2 - human variant 3	Yes	Reporter (p53_Luc)
Ral	HRas (G12V, E37G)	HRAS NM_001130442.1 - human variant 3	Not tested	
	Rgl2-CAAX	Rgl2 NM_009059.2 - mouse plus C-term KRAS	Not tested	
	RalA (G23V) (two forms - full and mature peptide)	RALA NM_005402.3 - human	Not tested	
CONTROLS	HcRed	N/A	N/A	
	Luciferase	N/A	N/A	
	MEK1	MAP2K1 NM_002755.3 - human	N/A	

MAPK, mitogen-activated protein kinase; ERK, extracellular-signal-regulated kinase; PI3K, phosphoinositide 3-kinase; AKT, protein kinase B; mTOR, mammalian target of rapamycin; Rheb, ras homolog enriched in brain; NF-κB, nuclear factor kappa-light-chain-enhancer of activated B cells; IKKα, inhibitor of nuclear factor kappa-B kinase subunit alpha; IKKβ, inhibitor of nuclear factor kappa-B kinase subunit beta; GSK3β, glycogen synthase kinase 3 beta; TCF-LEF, transcription factor-lymphoid enhancer-binding factor; JNK, jun N-terminal kinase; MEK, mitogen-activated protein kinase kinase; HES1, hairy and enhancer of split-1; MKK6, mitogen-activated protein kinase kinase 6; Smo, smoothened; Gli, glioma-associated oncogene; TGFβ, transforming growth factor beta; Smad, mothers against DPP homolog; BCL, B-cell lymphoma; ERα, estrogen receptor alpha; ERE, estrogen response element; AR, androgen receptor; ARE, androgen response element; YAP, yes-associated protein; LATS, large tumor suppressor kinase; Ral, RAS-like protein; Rgl2, ral guanine nucleotide dissociation stimulator-like 2
